# Minimum Wage Policies and Obstetric Disorders in the U.S.

**DOI:** 10.1016/j.amepre.2025.108156

**Published:** 2025-10-19

**Authors:** Mark E. McGovern, Slawa Rokicki, Hyunji Ahn, Nancy E. Reichman

**Affiliations:** 1 Department of Health Behavior, Society, and Policy, Rutgers School of Public Health, Piscataway, New Jersey;; 2 Department of Economics, Princeton University, Princeton, New Jersey;; 3 Department of Pediatrics, Rutgers Robert Wood Johnson Medical School, New Brunswick, New Jersey;; 4 Child Health Institute of New Jersey, Rutgers Robert Wood Johnson Medical School, New Brunswick, New Jersey

## Abstract

**Introduction:**

Hypertensive disorders of pregnancy are a major public health problem in the U.S., particularly given higher rates among disadvantaged communities. This study examined the impact of minimum wage policies on hypertensive and other obstetric disorders in a population-based setting.

**Methods:**

This analysis used U.S. national, state-level data from the 1992–2019 Global Burden of Disease study to estimate the associations between changes in state-level minimum wages and the incidence of maternal hypertensive and other obstetric disorders. Generalized difference-in-differences models were implemented. Analysis was performed in January–July 2025.

**Results:**

The mean incidence of maternal hypertensive disorders was 410 cases per 100,000 women. In fully adjusted models, a $1 or greater increase in the minimum wage was associated with a reduction in the incidence of maternal hypertensive disorders of 64.1 per 100,000 women (95% CI= −108.6, −19.7) over 5 years. Results were consistent across a variety of estimation strategies, including 2-way fixed effects and alternative approaches designed to account for staggered policy implementation.

**Conclusions:**

Findings suggest that minimum wage policies may play a role in reducing maternal hypertensive disorders. Further research is needed using individual-level data to explore effect heterogeneity and examine subgroup impacts, especially by race and ethnicity.

## INTRODUCTION

New-onset hypertensive disorders of pregnancy, including gestational hypertension, preeclampsia, and eclampsia, are leading contributors to maternal mortality and morbidity.^1^ Between 2007 and 2019, rates of maternal hypertensive disorders in the U.S. doubled to 8% of all pregnant women.^1^ Moreover, these conditions are characterized by substantial inequities; non-Hispanic Black women have rates of pregnancy-associated hypertension 1.3 times higher than non-Hispanic White women.^2^ Maternal hypertensive disorders have significant short- and long-run consequences, including increased risk of preterm birth, postpartum readmission, maternal mortality, and later-life cardiovascular disease.^3–5^ Although not as prevalent, other obstetric complications, such as maternal sepsis, are also associated with adverse maternal outcomes, including increased risk of mortality.^6^

Although the causes of obstetric disorders are multifaceted, the existing literature shows that structural and social determinants of health such as economic security play critical roles.^3,7–9^ Women living in lower-income areas have significantly higher rates of hypertensive disorders of pregnancy than women living in higherincome areas, and poverty-related stress is a likely contributor.^2,3,10–12^ Women in households with lower income are also less likely to have health insurance and more likely to have inadequate prenatal care.^13^ They are also more prone to experiencing poor nutrition, to smoke, and to have 3 or more chronic diseases, all of which are risk factors for hypertensive and other obstetric disorders.^2,5,14^ Members of racial and ethnic minority groups are disproportionately represented among those living in poverty.^15^

Minimum wage (MW) policies have potential to raise the incomes of millions of women, particularly those experiencing socioeconomic disadvantage who are more likely to earn the MW.^16,17^ Evidence from quasi-experimental studies indicates that higher MWs are associated with improved birth outcomes, including reductions in preterm birth and low birth weight^18–20^; improved mental health and reduced stress, including in the preconception period^21,22^; and reduced smoking and food insecurity.^23–25^ However, the authors are unaware of any studies to date that have rigorously examined the impacts of MWs on maternal hypertension or other obstetric disorders that may lie on the pathway between pregnancy and birth outcomes and affect subsequent maternal and infant health.^26^ The objective of this study was to assess the extent to which MW policies affect maternal health by quantifying potential impacts of MW increases on the incidence of maternal hypertensive and other obstetric disorders (specifically, those related to abortion and miscarriage, ectopic pregnancy, obstructed labor and uterine rupture, maternal hemorrhage, and maternal sepsis and other maternal infections) using quasi-experimental methods to account for unobserved potential confounders.

The study hypothesis was that increases in MWs would reduce incidence of maternal disorders, with strongest effects on hypertensive-related disorders because of the well-established link between hypertension and social determinants of health.^3,10,27^ To allow enough time for MW changes to affect health through pathways such as reduced financial stress, effects were estimated over a 5-year window.

## METHODS

### Study Sample

In this retrospective population-based study, aggregate annual state-level longitudinal data from the Global Burden of Disease (GBD) study were analyzed. The study period was from 1992 to 2019, and the inclusion criteria were all 50 states and Washington, DC for all years of the study period. There were no exclusion criteria. The sample size thus included 1,428 state-year observations, representing all 50 U.S. states and Washington, DC across 28 years (51 × 28=1,428), with no missing data.

To examine effects of changes in state-level MWs on maternal disorders, MW data were obtained from the University of Kentucky Center for Poverty Research (UKCPR) and linked by state and year to maternal disorder incidence rates obtained from the GBD. Additional data on other state-level economic and policy factors were obtained from UKCPR, and data on state Medicaid policies were obtained from Kaiser Family Foundation.^28^ Data on birth and population estimates by race and ethnicity were obtained from the Centers for Disease Control and Prevention and Integrated Public Use Microdata Series.^29^

The GBD study has been widely used to assess levels and trends of burden of diseases, injuries, and risk factors around the world,^30,31^ including within the U.S.^32^ Compared to other data sources for quantifying maternal morbidities, GBD has advantages. First, GBD methodology synthesizes a variety of data sources to produce health estimates and uses standardized procedures to estimate the incidence and prevalence of disease. Statistical code for GBD estimation is publicly available.^32^ Sources for nonfatal maternal disorders data for the U.S. were clinical data, including hospital discharge data and commercial claims (Marketscan database), as well as Centers for Disease Control and Prevention surveillance data.^32–34^ In comparison, other data sources, such as birth records, have been found to significantly underreport complications of pregnancy.^35^ Second, GBD systematically accounts for differences in data sources and biases and analyzes levels and trends for causes and risk factors within the same computational framework. This maximises comparability across states and time, which is critical for this study’s analysis across 51 states (including DC) and 28 years.^32^ In contrast, although some maternal health measures are available through the State Inpatient Databases provided by Agency for Healthcare Research and Quality’s Healthcare Cost and Utilization Project, data are only available for a limited number of states, particularly in the 1990s.

### Measures

State MW information was recorded in the UKCPR data as a continuous variable indicating the MW in each state for January 1 of each year. For states that did not have MWs that were higher than the federal level, the federal MW for that year was used. Appendix Figure 1 shows the evolution of the federal MW and the MW in states that set their own laws, over the course of the study period. In the main analysis, this continuous variable was dichotomized into a binary variable indicating sufficiently large MW increases, defined as a year-to-year increase of $1 or more. The $1 cutoff was chosen to be in line with previous literature and provide a policy-relevant interpretation.^36^ The distribution of MW changes is shown in Appendix Figure 2 (available online). About 6% of year-to-year MW changes were at least $1. State-years that experienced a MW increase of $1 or more relative to the previous year in that state were classified as exposed. State-years that did not experience such an increase were classified as unexposed.

Outcomes were nonfatal maternal disorders due to direct obstetric complications, grouped into the following categories: (1) maternal hypertensive disorders, (2) abortion and miscarriage, (3) ectopic pregnancy, (4) obstructed labor and uterine rupture, (5) maternal hemorrhage, and (6) maternal sepsis and other maternal infections, as well as an overall measure of any maternal disorder. This list reflects the subcategories of nonfatal maternal disorders available in the GBD data. The analysis focused on measures of nonfatal maternal disorders rather than composite measures such as disability-adjusted life years that integrate mortality with morbidity because changes in state-level reporting of maternal deaths make interpreting variation over time challenging in the U.S.^37^

Obstetric disorder outcomes were measured as yearly age-standardized incidence per 100,000 of the female population within each state. In sensitivity analysis, measures of prevalence of maternal disorders were examined.

State-year controls were used unmodified from their sources, and the following continuous variables were included and lagged 2 years: the state Earned Income Tax Credit rate; maximum monthly Temporary Assistance to Needy Families and Supplementary Nutrition Assistance Program benefit for a 2-person family; unemployment rate; poverty rate; state gross product; population size; number of births; and the percentage of the population that was Asian, American Indian/Alaska Native, non-Hispanic Black, and non-Hispanic White (with Hispanic as the reference). Finally, a binary indicator for whether Medicaid was expanded under the 2010 Affordable Care Act was also included.

### Statistical Analysis

First, descriptive analyses were conducted examining differences in outcome and control variables between state-years with MWs equal to the federal MW and state-years with MWs higher than the federal MW. Then, linear regression was used to model the association of outcome measures with MW increases, conditional on covariates. Following recent advances in the causal inference literature, the impact of MW increases was assessed in an event study framework using a generalized difference-in-differences (DD) 2-way fixed effects (TWFE) approach.^38,39^

For each MW change event, an analysis window of 9 years (4 years prior to and after the event year) was defined, and outcomes for all state-years falling within this window were evaluated.^38^ A 4-year postperiod analysis was chosen to be consistent with previous work that finds that effects of MW changes on health may take several years to appear and because this is consistent with possible effect pathways, including reduced stress, improved access to care, and improved nutrition, which are likely to require time to ultimately affect risk of maternal hypertensive disorders.^40–42^ Because the data are a long panel with relatively few states, the endpoints of the effect window were binned at 5 years. In robustness checks, the assumption that treatment effects are likely to be the same beyond the window was assessed by implementing a model with 10 preperiods and 10 postperiods, finding that effect estimates remained consistent 5 years after an event (Appendix Figure 3, available online). Models were adjusted for state- and timefixed effects, state-specific time trends, and state-year level controls. A total treatment effect for the impact of a $1 or greater MW increase over Years 0–4 was estimated. In all analyses, SEs were clustered at the state level.^43^ Analysis was performed in January–July 2025 using Stata 18. Additional statistical model details are shown in the Appendix Material (available online). Several robustness checks were conducted, including varying the outcome using prevalence measures instead of incidence, varying the exposure by defining an event as a MW increase of $0.75 or more, and restricting the study time period to more recent years.

In addition, recent research raises concerns about the consistency of TWFE estimates when treatments are adopted in a staggered way. Specifically, TWFE estimates have been shown to produce consistent estimates only when treatment effects are homogenous across time and groups. To address this concern, results were replicated using estimators robust to heterogeneous treatment effects, including Callaway and Sant’Anna (2021), de Chaisemartin and D’Haultfúuille (2020), and Sun and Abraham (2021).^44–46^ This analysis was deemed nonhuman-subjects research and exempt from full review under Rutgers IRB.

## RESULTS

Of 1,428 state-years in the analytical data set, in 915 (64%) the MW was equal to the federal MW, whereas in 513 (36%) the MW was above the federal minimum (Table 1). State characteristics varied across these groups, with the state Earned Income Tax Credit rate, the maximum Temporary Assistance to Needy Families/Supplementary Nutrition Assistance Program benefit, and likelihood of Medicaid expansion being substantially higher in state-years with higher than federal MWs. The mean incidence of maternal hypertensive disorders was higher in state-years where the federal MW applied, with a mean of 431 cases per 100,000 women in state-years with the federal MW, compared with a mean of 372 cases per 100,000 women in state-years with MWs higher than the federal rate. Incidence of maternal obstructed labor and maternal sepsis was also lower for state-years with higher than federal MWs, whereas incidence of maternal abortion was higher.

In total, there were 61 MW events (increases of $1 or greater) during the study period. Table 2 shows the overall impact of a MW change of $1 or greater on incidence of maternal disorders, overall and by subcategory, totalled over the 0–4 years after the change. For all outcomes, estimates for each time point in the 9-year window are shown in Appendix Table 1 (available online). A $1 or greater MW change was associated with a reduction in incidence of maternal hypertensive disorders of 64.8 per 100,000 women (95% CI= −109.5, −20.0) over 5 years. There was also an association with reduced incidence of maternal hemorrhage (−27.4 per 100,000; 95% CI= −51.8, −3.1). There were no significant associations with other subcategories of obstetric disorders or all maternal disorders. Sensitivity analyses are shown in Appendix Table 2 (available online), with consistent results for prevalence measures, exposure of $0.75 or more, and restricted time period to 2000–2019 for maternal hypertension. However, when data were restricted to 2010–2019, estimates were null for all outcomes, although sample sizes were reduced substantially. Results for maternal hemorrhage were more sensitive to robustness checks, with null findings for events of $0.75 and both time period restrictions. Results were also robust to omitting controls for a number of births and population size.

**Figure 1.**
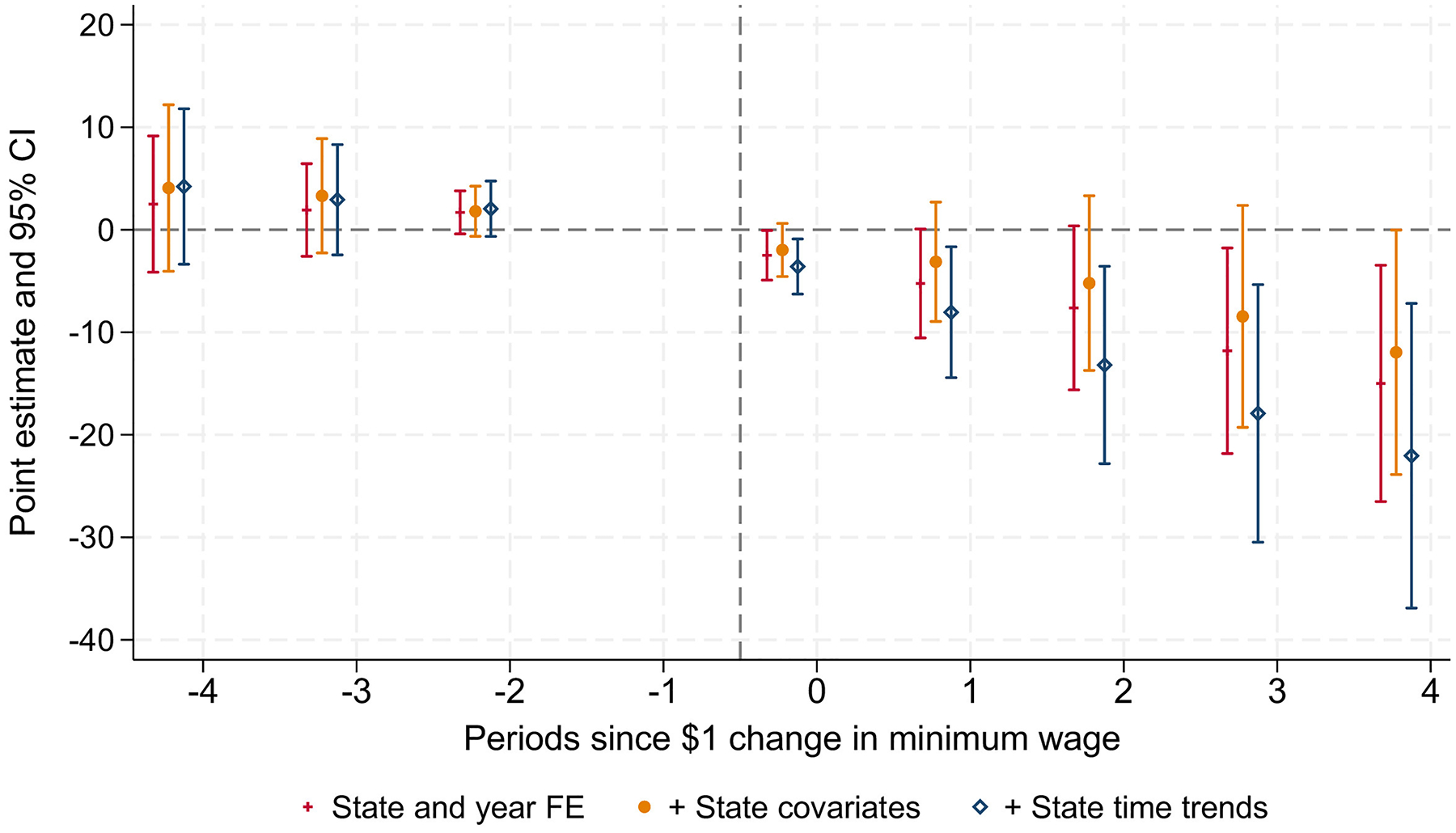
Estimates of the impact of a $1 or greater minimum wage increase on the incidence of maternal hypertensive disorders. *Note*: Point estimates from 2-way fixed effect linear models for the incidence of maternal hypertensive disorders (age standardized per 100,000 of the female population) are shown. Controls are added sequentially, first in a model with time- and state-fixed effects, second in a model that additionally controls for state covariates listed in the footnotes of Table 2, and third, in a model that additionally controls for state-specific time trends.

Estimates from DD models using the TWFE event study approach for maternal hypertensive disorders are shown in Figure 1, with controls added sequentially to observe sensitivity to covariates. State-level covariates and state time trends did not substantially change the estimates. Results showed a downward trend in the postperiod, with the largest negative impacts 2–4 years after the MW event.

**Figure 2.**
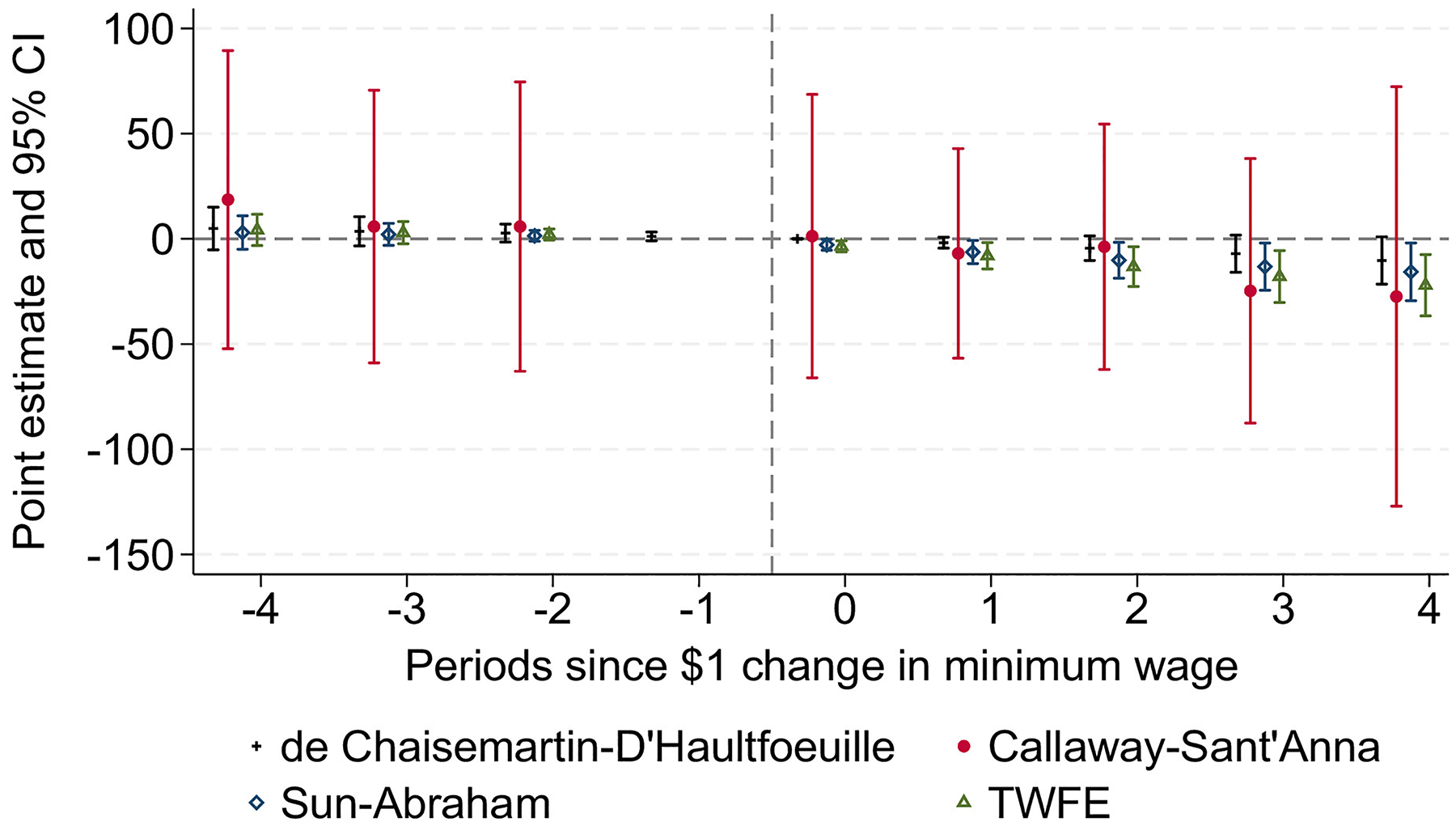
Estimates of the impact of a $1 or greater minimum wage increase on the incidence of maternal hypertensive disorders using alternative heterogeneity-robust estimators. *Note*: Point estimates are from linear models using 3 heterogeneity robust estimators.^45–47^ TWFE estimates are also shown for comparison. In addition to state- and year-fixed effects, all models are also adjusted using the covariates listed in the footnotes of Table 2. TWFE, 2-way fixed effects.

Results of estimators robust to heterogeneous treatment effects are shown in Figure 2 (estimates are reported in Appendix Table 3, available online).^45–47^ Overall, these were similar across the various specifications. Although the Callaway and Sant’Anna^45^ estimator may be less efficient and can lead to wider CIs, results from these alternative models align with the TWFE estimates in Figure 2.

## DISCUSSION

This study found that increases in MWs of $1 or more were associated with meaningful reductions in the incidence of maternal hypertensive disorders at the state level. Results were consistent for both TWFE models and newer, alternative estimators that are robust to staggered treatment adoption. The magnitude of the association was larger in the 2–4 years after the MW change, suggesting a causal pathway through improved preconception or interconception health rather than through pregnancy health. This is consistent with maternal hypertensive disorders having preconception origins, in which modifiable risk factors such as poor nutrition, stress, and depression before pregnancy contribute to increased risk of hypertension during pregnancy.^48,49^ Findings also suggest that MWs were associated with reductions in postpartum hemorrhage; however, those results were more sensitive to model specification. Results for the association between MWs and other subcategories and all maternal disorders were not statistically significant once adjusted for confounders.

Rates of hypertensive disorders of pregnancy have doubled in the U.S. in recent decades and are one of the leading causes of maternal mortality.^1^ By addressing social determinants of health, MWs may positively affect pathways affecting risk factors for hypertension.^10^ Previous research has found that higher MWs reduce the likelihood of stressful life events before and during pregnancy,^21^ and impacts estimated in this study are consistent with pathways linking material resources with stress and health through, for example, increased allostatic load and depressive symptoms.^50,51^ The cardiovascular system has been found to be affected by the physiologic consequences of chronic exposure to repeated or prolonged stress.^52^ In addition, by lowering poverty rates, higher MWs may improve healthy behaviours such as diet and physical activity, and reduce smoking.^23,41^ Finally, higher MWs have also been linked to increased healthcare access, which may improve management of chronic diseases present before pregnancy.^5,53^

Results from this study are consistent with those of 3 previous studies in the general population that found significant associations between MWs and reduced hypertension prevalence, but to the authors’ knowledge, this is the first to examine maternal hypertensive disorders, specifically.^40,54,55^ In addition, findings are consistent with some previously observed associations between state MWs and improved infant health outcomes, and provide evidence for a potential mechanism underlying the link between MWs and adverse birth outcomes such as preterm birth.^20^ More broadly, findings provide further evidence on the impact of economic policies on maternal health, consistent with recent studies that have shown that social safety net programs and tax credits for lower-income families have positive benefits for maternal health.^56–59^

This study has a number of potential policy implications. Although the federal MW has not changed since 2009, states are increasingly adopting their own policies, therefore changes to MWs are feasible at subnational levels. In 2019, 30 states had MW rates higher than the federal rate, compared with only 10 in 2010.^17^ Yet, there are approximately 127 million Americans living in states where the current federal minimum of $7.25 applies. Raising this to $15 per hour would increase the earnings of 32 million workers, who are more likely to be women and from communities of color, and this study shows the potential for these policies to improve maternal health.^16,17^ Further research is needed using individual-level data to explore effect heterogeneity and examine subgroup impacts by race and ethnicity.

This study has several strengths, including applying recent advances in causal inference to national-level data on maternal health from all 50 states and Washington, DC, over a 30-year period to address important substantive and policy relevant questions. The GBD data were collected and compiled using a standardized process designed to provide estimates of the disease burden through a transparent and validated methodology. Under the assumption that outcomes would evolve in a parallel way in the absence of MW changes, a generalized DD approach rules out various confounders that could bias the results, including time-varying observed confounders, time-invariant state-level unobserved confounders, and common trends across states. Using an event study framework, this study examined impacts of MW increases up to 4 years later, and results confirm that it is important to consider effects beyond the year of implementation. The event study framework also provided support for the parallel trends assumption by showing no evidence of pretrends in the 4 years prior to MW changes. Finally, consistent results were found using alternative estimators that address limitations of the TWFE approach.^45–47^

### Limitations

This study also has limitations. First, data were aggregated at the state-level. Despite using rigorous quasi-experimental statistical methods and a variety of robustness checks, this study cannot definitively rule out the possibility that the estimates reflect time-varying unobserved confounders. In addition, the effects of MW on specific populations, such as Black women, known to be more at risk of adverse maternal outcomes, were not able to be assessed with the data.^2^ Given that these populations are also more likely to be earning the MW, this is a priority for future research. Third, this study focused on obstetric disorders associated with pregnancy that are available in the GBD study. A priority for future research is to use individual-level data to trace the impact of MW policies from the preconception period through postpartum period, and to examine a wider range of health outcomes. Finally, although GBD data are consistently measured, the underlying records used within each state may not be free from measurement error, missing information, or other data quality issues. Combined with the modeling process used by the GBD study and the potential implications for inference and estimates of parameter uncertainty, this highlights the need for individual-level analyses with more directly observed outcomes.

## CONCLUSIONS

The findings from this study suggest that increases in MWs are associated with reductions in the incidence of maternal hypertensive disorders, which in the U.S. is of particular importance given increases in the proportion of pregnant women who are experiencing this outcome. Results suggest that raising federal- or state-level MWs may have important maternal health benefits.

## Supplementary Material

Appendix

Supplemental materials associated with this article can be found in the online version at https://doi.org/10.1016/j.amepre.2025.108156.

## Figures and Tables

**Table 1.** Descriptive Statistics of State-Years in Analytical Sample

Variables	State-years at federal MW, mean (SD or %)	State-years at higher than federal MW, mean (SD or %)	Total, mean (SD or %)
*n*	915 (64.1%)	513 (35.9%)	1,428 (100%)
State MW (U.S. Dollars)	5.37 (1.31)	7.48 (1.67)	6.13 (1.77)
State EITC rate	3.46 (8.02)	10.02 (13.83)	5.82 (10.94)
TANF/SNAP 2-person benefit	573.3 (139.1)	742.0 (158.2)	633.9 (167.1)
Medicaid expanded under ACA, *n* (%)	43 (4.7%)	144 (28.1%)	187 (13.1%)
Population	5,630,566 (5,463,737)	6,035,248 (7,993,638)	5,775,945 (6,487,271)
Gross state product	219,720.6 (248,755.6)	320,140.9 (462,266.6)	255,796.0 (344,424.0)
Unemployment rate	5.30 (1.75)	5.74 (2.05)	5.46 (1.88)
Poverty rate	13.07 (3.75)	11.94 (3.15)	12.67 (3.59)
Number of births	79,250 (83,476)	77,251 (108,613)	78,532 (93,257)
Percentage AIAN	1.5 (2.4)	1.9 (3.9)	1.7 (3.0)
Percentage Asian	2.5 (4.7)	6.9 (12.3)	4.1 (8.5)
Percentage NH Black	12.0 (10.7)	10.0 (12.1)	11.3 (11.3)
Percentage Hispanic	7.8 (8.6)	11.7 (10.4)	9.2 (9.5)
Percentage NH White	76.2 (13.7)	69.4 (19.1)	73.8 (16.1)
Incidence of maternal hypertensive disorders	431.2 (63.1)	372.3 (74.2)	410.1 (73.0)
Incidence of maternal abortion and miscarriage	521.7 (241.8)	568.0 (268.3)	538.3 (252.5)
Incidence of ectopic pregnancy	97.5 (26.3)	90.4 (29.5)	94.9 (27.7)
Incidence of maternal obstructed labor and uterine rupture	479.8 (143.2)	375.8 (131.7)	442.4 (147.8)
Incidence of maternal hemorrhage	235.6 (44.1)	222.6 (48.8)	230.9 (46.3)
Incidence of maternal sepsis and other maternal infections	592.2 (149.4)	528.1 (129.7)	569.2 (145.9)
Incidence of all maternal disorders	2,358.0 (474.2)	2,157.2 (501.9)	2,285.9 (493.7)

*Note*: Data presented are mean (SD), unless specified otherwise. Data are from GBD, 1992–2019; UKCPR, 1992–2019; KFF, 2014–2019; and CDC Wonder birth records, 1992–2019.

ACA, Affordable Care Act; AIAN, American Indian/Alaska Native; CDC, Centers for Disease Control and Prevention; EITC, Earned Income Tax Credit; GBD, Global Burden of Disease; MW, minimum wage; NH, non-Hispanic; SNAP, Supplementary Nutrition Assistance Program; TANF, Temporary Assistance for Needy Families; UKCPR, University of Kentucky Center for Poverty Research.

**Table 2:** Total Cumulative Impacts of $1 or More Increase in MW Over 0–4 Years

Impact estimate	Maternal hypertensive disorders	Abortion and miscarriage	Ectopic pregnancy	Obstructed labor and uterine rupture	Maternal hemorrhage	Maternal sepsis and other maternal infections	All maternal disorders
Estimate	−64.8***	−112.8	−0.7	46.4	−27.4*	−68.2	−227.5
95% CI	**(−109.5, −20.0)**	(−281.4, 55.8)	(−14.1, 12.7)	(−30.4, 123.3)	**(−51.8, −3.1)**	(−140.3, 3.9)	(−503.8, 48.7)
*n*	1,428	1,428	1,428	1,428	1,428	1,428	1,428

*Note:* Boldfaces indicate statistical significance (*p<0.05, **p<0.01, and ***p<0.005).

Estimates for the total MW impact 0–4 years after the MW change are shown with 95% CIs, derived from SEs that are clustered by state. Outcomes are age-standardized incidence of these conditions per 100,000 of the female population. Models adjust for state- and year-fixed effects, state-specific time trends, and the following covariates shown in Table 1: whether Medicaid was expanded under the 2010 Affordable Care Act; the state EITC rate; maximum monthly TANF and SNAP benefit for a 2-person family; unemployment rate; poverty rate; state gross product; population size; number of births; and the percentage of the population that was Asian, American Indian/Alaska Native, non-Hispanic Black, and non-Hispanic White (with Hispanic as the reference).

EITC, Earned Income Tax Credit; MW, minimum wage; TANF, Temporary Assistance for Needy Families.
