# Supplementary material for: Minimum Wage Policies and Obstetric Disorders in the U.S
Source: Am J Prev Med. Author manuscript; Available in PMC 2026 Mar 16. (PMC12990581; doi:10.1016/j.amepre.2025.108156)
Supplement: Appendix [file NIHMS2153312-supplement-Appendix.pdf]

Minimum wage policies and obstetric disorders in the U.S.

October 2025

Mark E. McGovern[*], PhD, Slawa Rokicki[*], PhD, Hyunji Ahn, PhD,

Nancy Reichman, PhD

[*]Joint first authors

American Journal of Preventative Medicine

Appendix Online Material

Appendix Material: Statistical Model

The linear predictor representation of the TWFE DD model in this event study framework is as follows:

$$Y_{st} = \alpha + \beta_1 MW(t-4)_{s,t} + \beta_2 MW(t-3)_{s,t} + \beta_3 MW(t-2)_{s,t} + \beta_4 MW(t)_{s,t} + \beta_5 MW(t+1)_{s,t} + \beta_6 MW(t+2)_{s,t} + \beta_7 MW(t+3)_{s,t} + \beta_8 MW(t+4)_{s,t} + \delta_1 MW(t<-4)_{s,t} + \delta_2 MW(t>4)_{s,t} + Z_{s,t-2}\beta_2 + \theta_t + \mu_s + \varepsilon_{ist} \qquad (Eq. S1)$$

Where Y_{st} is the outcome for state s in year t. In this model, events are stacked across states so that the years are normalized to represent the i years before and after the MW increases. $MW(t-i)_{s,t}$ are indicator variables that take the value 1 for the ith year before or after a MW change of $1 or more in state s. We omit year $t-1$ as the reference category. Coefficients $\beta_4 - \beta_8$ quantify MW impacts on the outcome zero to four years after the event, while coefficients $\beta_1 - \beta_3$ assess pre-trends in our outcomes. In a well-specified model that meets the main DD identifying assumption of parallel trends, we would expect coefficients $\beta_1 - \beta_3$ to be close to 0 after adjusting for covariates. In contrast, given our hypothesis that MWs

improve maternal health, we would expect to see negative coefficients on coefficients $\beta_4 - \beta_8$. $MW(t < -4)_{s,t}$ and $MW(t > 4)_{s,t}$ are binning indicator variables that take the value 1 for periods that are more than 4 years before a MW change, and more than 4 years after a MW change, respectively.

θ_t is a vector of year fixed effects to control for time-dependent factors common across states. μ_s is a vector of state fixed effects and accounts for all state-level time-invariant unobserved factors. ε_{ist} is the error term. Z_{st-2} is a vector of time-varying state-level control variables, which include measures of generosity of other state-level welfare policies lagged by two years to ensure they were not on the causal pathway, including whether Medicaid was expanded under the 2010 Affordable Care Act, the state EITC rate, maximum monthly TANF and SNAP benefit for a 2-person family, unemployment rate, poverty rate, state gross product, population size, number of births, and the percentage of the population that was Asian, American Indian/Alaska Native, non-Hispanic Black, and non-Hispanic White (with Hispanic as the reference). These controls address concerns that various state-by-year factors may confound the relationship between MW policies and maternal health outcomes. We also adjusted for state-specific time trends, which further control for unaccounted factors that vary by state over time. All covariates are continuous except for a binary indicator denoting whether the state had expanded Medicaid.

In this type of event study analysis, observations are defined by their position in time relative to the occurrence of treatment events. In this paper, we construct, for each state-year, a time variable, known in the literature as “event time” that measures their proximity to MW increases of \$1 or more in that particular state. For some states, there are no treatment events and therefore no treated units. We then use this event time to model changes in our outcome before and after the occurrence of treatment events. The traditional DD and event study frameworks are closely related, in that the average of event study effect estimates in the post period should

correspond to traditional DD estimates. However, event studies facilitate exploration of how treatment effects evolve across time relative to the MW event, and also provide an assessment of pre-treatment trends that have important implications for internal validity. As shown in Appendix Figure 1, the federal MW rose from $4.25 in 1992 to $7.25 in 2009, where it has remained. In state-years which had MW policies set to higher than the federal MW, the mean MW has risen from $4.67 to $10.44.

Appendix Material: Additional Results

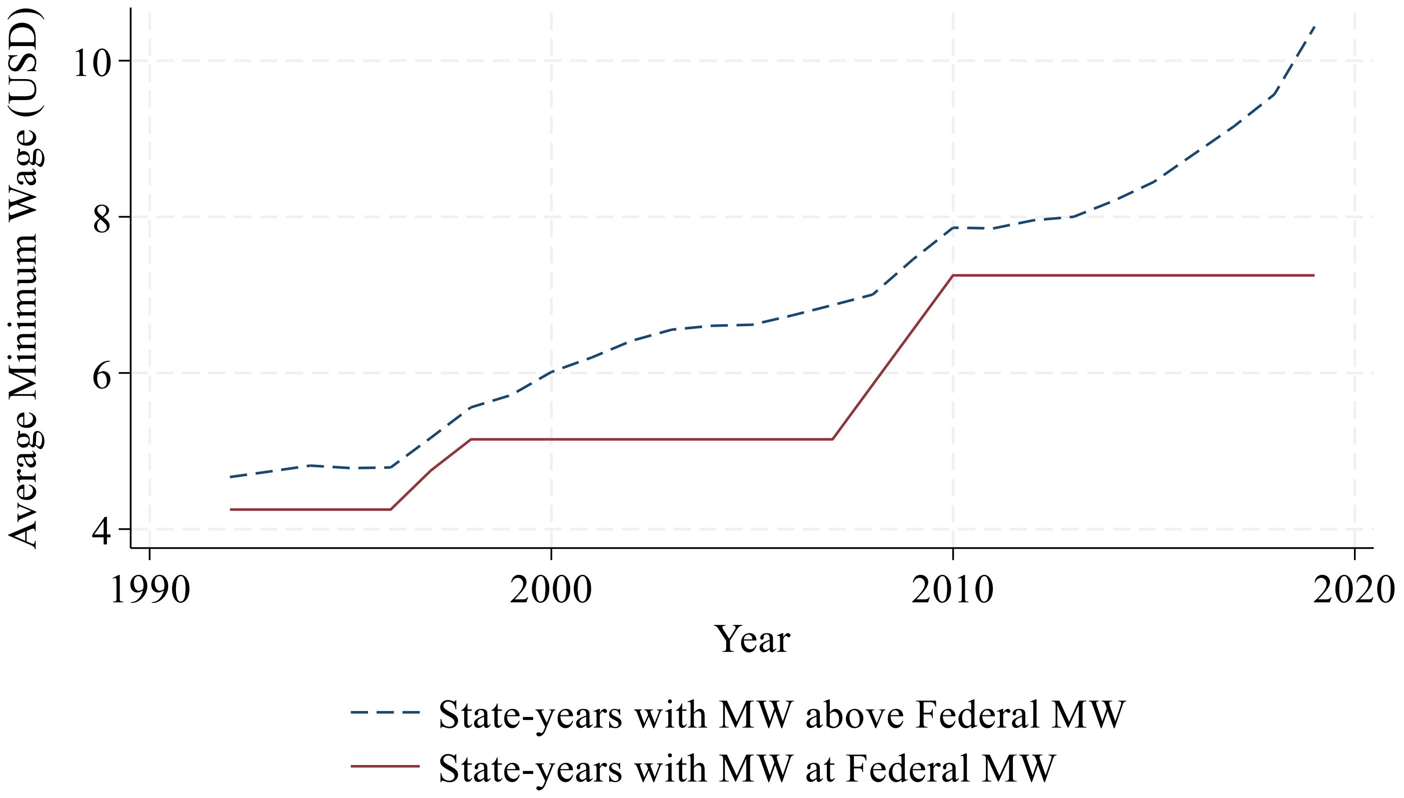

Appendix Figure 1. Average minimum wage in state-years with minimum wages above the federal minimum wage and state-years with minimum wage equal to federal minimum wage

Note: Data are from UKCPR 1992-2019.

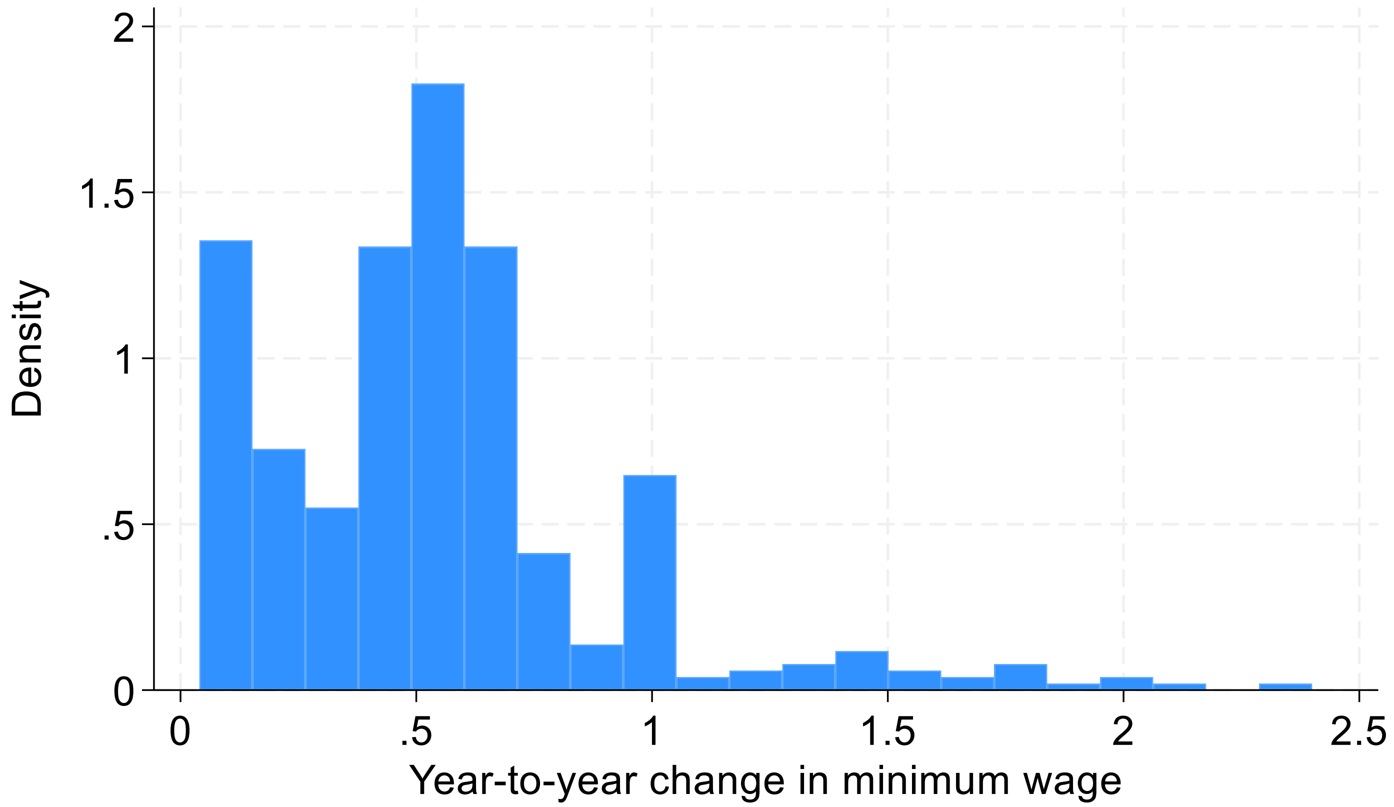

Appendix Figure 2. Distribution of minimum wage changes (USD)

Note: Figure shows distribution of changes in states' minimum wages (USD) between consecutive years for all minimum wage changes that are greater than 0.

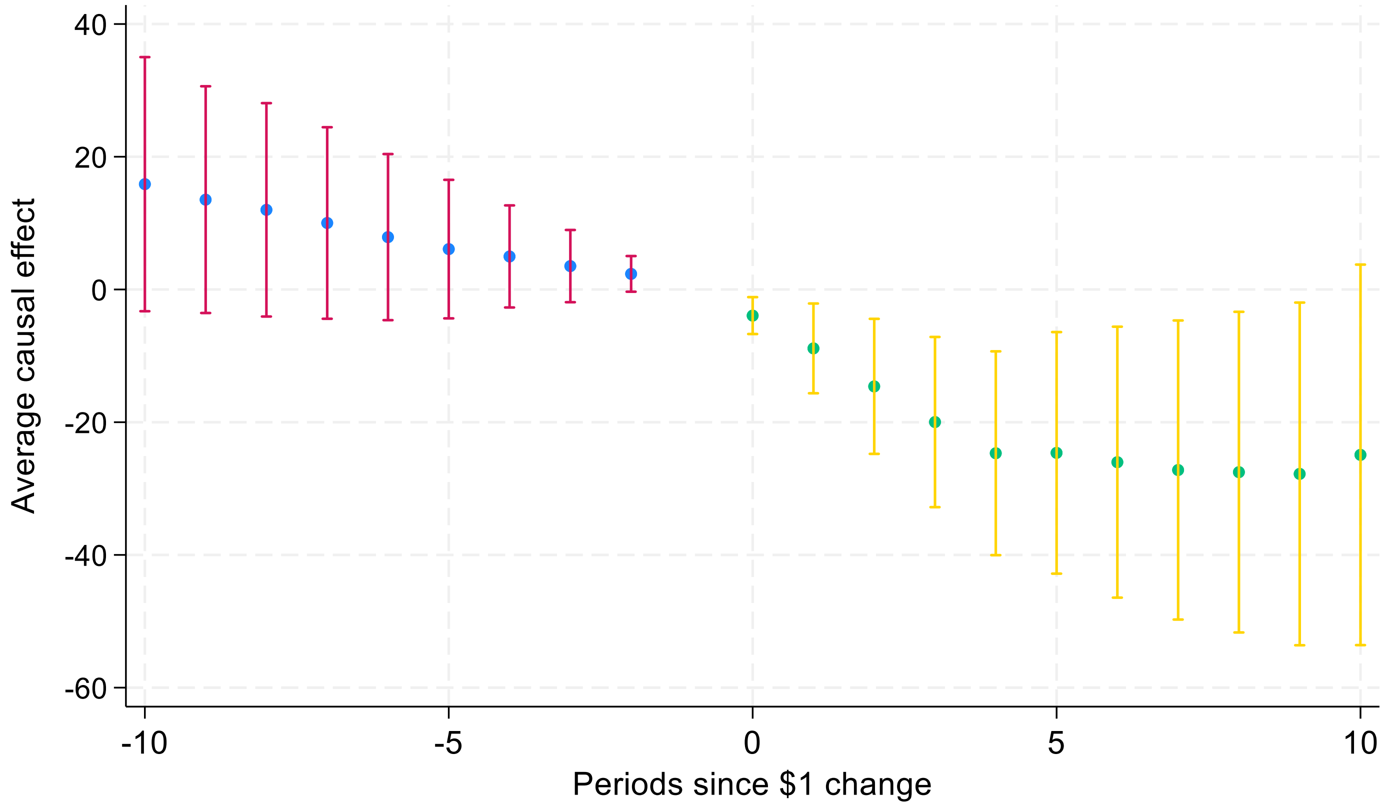

Appendix Figure 3. Estimates of the impact of a $1 or greater minimum wage increase on the incidence of maternal hypertensive disorders, with 10 years of leads and lags

Note: Point estimates from two-way fixed effect linear models for the incidence of maternal hypertensive disorders (age-standardized per 100,000 of the female population) are shown in a model that includes 10 years of leads and 10 years of lags. Model includes full set of controls.

Appendix Table 1 Regression results for unadjusted and incrementally adjusted two-way fixed effects models

Table 1a. Maternal hypertensive disorders

	(1)<br>State & year FE	(2)<br>+Covariates	(3)<br>+Time trends
T-5 or before	0.541	3.973	8.983
	[-14.736,15.818]	[-10.724,18.669]	[-3.112,21.078]
T-4	2.505	4.074	4.218
	[-4.305,9.315]	[-4.246,12.394]	[-3.548,11.983]
T-3	1.929	3.312	2.929
	[-2.699,6.556]	[-2.399,9.023]	[-2.582,8.441]
T-2	1.694	1.804	2.049
	[-0.455,3.844]	[-0.704,4.311]	[-0.720,4.818]
T-1 [ref]			
T	-2.488*	-1.972	-3.590*
	[-4.970,-0.005]	[-4.625,0.682]	[-6.345,-0.835]
T+1	-5.237	-3.122	-8.045*
	[-10.683,0.209]	[-9.089,2.845]	[-14.586,-1.504]
T+2	-7.623	-5.202	-13.192**
	[-15.817,0.571]	[-13.929,3.526]	[-23.057,-3.327]
T+3	-11.801*	-8.451	-17.913**
	[-22.078,-1.523]	[-19.542,2.641]	[-30.791,-5.035]
T+4	-14.987*	-11.950	-22.044**
	[-26.802,-3.171]	[-24.170,0.269]	[-37.271,-6.816]
T+5 or after	-17.433	-15.180	-21.404*
	[-35.391,0.525]	[-33.287,2.927]	[-41.300,-1.507]

Table 1b. Abortion and miscarriage:

	(1)<br>+State & year FE	(2)<br>+Covariates	(3)<br>+Time trends
T-5 or before	-6.893<br>[-122.512,108.726]	4.073<br>[-41.928,50.073]	6.822<br>[-29.839,43.484]
T-4	-37.492<br>[-119.112,44.128]	-24.973<br>[-51.660,1.713]	-6.143<br>[-20.628,8.343]
T-3	-37.164<br>[-112.445,38.118]	-22.935<br>[-46.532,0.661]	-6.900<br>[-17.876,4.075]
T-2	-1.067<br>[-7.021,4.887]	-5.068<br>[-12.152,2.015]	-1.501<br>[-9.176,6.174]
T-1 [ref]			
T	0.485<br>[-6.660,7.630]	-0.657<br>[-7.809,6.495]	-2.887<br>[-11.811,6.038]
T+1	17.972<br>[-18.388,54.332]	7.819<br>[-14.108,29.745]	-11.744<br>[-33.433,9.945]
T+2	21.307<br>[-24.165,66.779]	6.236<br>[-27.915,40.388]	-22.072<br>[-56.486,12.341]
T+3	19.543<br>[-35.354,74.440]	5.647<br>[-36.583,47.878]	-32.998<br>[-79.980,13.984]
T+4	11.803<br>[-52.422,76.028]	4.463<br>[-46.944,55.869]	-43.097<br>[-106.095,19.901]
T+5 or after	-40.731<br>[-192.145,110.683]	14.073<br>[-80.109,108.254]	-75.129<br>[-200.590,50.332]

Table 1c. Ectopic pregnancy

	(1)<br>+State & year FE	(2)<br>+Covariates	(3)<br>+Time trends
T-5 or before	3.624<br>[-0.596,7.844]	1.751<br>[-2.235,5.737]	2.442<br>[-0.556,5.439]
T-4	1.509<br>[-0.451,3.468]	0.229<br>[-1.812,2.270]	0.458<br>[-1.348,2.264]
T-3	0.833<br>[-0.550,2.216]	-0.078<br>[-1.652,1.495]	0.017<br>[-1.335,1.368]
T-2	0.365<br>[-0.357,1.087]	-0.075<br>[-0.768,0.617]	0.192<br>[-0.512,0.896]
T-1 [ref]			
T	-0.138<br>[-0.918,0.643]	0.112<br>[-0.706,0.929]	-0.131<br>[-0.954,0.692]
T+1	-0.099<br>[-1.688,1.490]	0.076<br>[-1.633,1.785]	-0.304<br>[-2.096,1.488]
T+2	0.334<br>[-2.008,2.676]	0.622<br>[-1.918,3.162]	-0.117<br>[-2.878,2.644]
T+3	0.047<br>[-2.969,3.062]	0.851<br>[-2.392,4.094]	-0.082<br>[-3.846,3.683]
T+4	-0.473<br>[-4.182,3.236]	0.507<br>[-3.371,4.386]	-0.093<br>[-5.011,4.825]
T+5 or after	-1.207<br>[-6.356,3.943]	0.202<br>[-4.785,5.190]	-0.398<br>[-8.273,7.476]

Table 1d. Obstructed labor and uterine rupture

	(1)<br>+State & year FE	(2)<br>+Covariates	(3)<br>+Time trends
T-5 or before	2.675	4.245	5.728
	[-17.315,22.665]	[-12.686,21.175]	[-9.525,20.981]
T-4	-0.646	-0.891	-2.890
	[-8.872,7.580]	[-8.088,6.306]	[-10.869,5.088]
T-3	-1.161	-1.633	-3.361
	[-7.414,5.091]	[-7.670,4.405]	[-10.221,3.499]
T-2	0.211	0.646	-0.636
	[-3.318,3.740]	[-3.365,4.658]	[-5.303,4.031]
T-1 [ref]			
T	0.566	2.267	2.314
	[-3.998,5.130]	[-2.306,6.841]	[-2.731,7.358]
T+1	1.252	6.767	7.083
	[-9.419,11.922]	[-2.847,16.382]	[-3.546,17.712]
T+2	5.891	12.538	11.589
	[-9.498,21.280]	[-2.855,27.932]	[-5.266,28.445]
T+3	5.011	13.140	12.352
	[-13.912,23.934]	[-5.717,31.998]	[-9.792,34.497]
T+4	3.918	11.389	13.086
	[-16.487,24.322]	[-9.024,31.802]	[-12.453,38.625]
T+5 or after	0.171	5.259	13.096
	[-26.906,27.248]	[-19.743,30.261]	[-18.955,45.147]

Table 1e. Maternal haemorrhage

	(1)<br>+State & year FE	(2)<br>+Covariates	(3)<br>+Time trends
T-5 or before	-4.725<br>[-12.810,3.359]	-5.018<br>[-13.590,3.553]	-1.554<br>[-10.395,7.287]
T-4	-1.607<br>[-5.436,2.223]	-2.422<br>[-6.947,2.103]	-1.029<br>[-5.509,3.450]
T-3	-1.145<br>[-3.608,1.319]	-1.681<br>[-4.916,1.554]	-0.936<br>[-3.968,2.095]
T-2	-0.085<br>[-1.196,1.026]	-0.458<br>[-1.788,0.872]	0.118<br>[-1.479,1.716]
T-1 [ref]			
T	-0.402<br>[-1.535,0.732]	0.044<br>[-1.191,1.278]	-0.924<br>[-2.408,0.560]
T+1	-1.345<br>[-4.169,1.478]	-0.010<br>[-2.996,2.976]	-2.677<br>[-6.032,0.678]
T+2	-2.394<br>[-6.520,1.733]	-0.689<br>[-4.817,3.440]	-4.942<br>[-9.935,0.051]
T+3	-4.323<br>[-10.115,1.469]	-2.198<br>[-7.673,3.277]	-7.882*<br>[-14.810,-0.954]
T+4	-6.995<br>[-14.522,0.531]	-4.556<br>[-11.590,2.477]	-11.012*<br>[-20.226,-1.797]
T+5 or after	-14.866***<br>[-24.481,-5.252]	-11.462*<br>[-21.543,-1.382]	-20.396**<br>[-35.203,-5.589]

Table 1f. Maternal sepsis and other maternal infections

	(1)<br>+State & year FE	(2)<br>+Covariates	(3)<br>+Time trends
T-5 or before	-11.950<br>[-29.995,6.096]	-11.474<br>[-30.585,7.636]	1.274<br>[-16.585,19.132]
T-4	-3.200<br>[-13.562,7.162]	-4.794<br>[-15.023,5.435]	-0.080<br>[-9.795,9.636]
T-3	-2.688<br>[-10.590,5.214]	-3.311<br>[-11.154,4.532]	-0.452<br>[-7.319,6.416]
T-2	0.769<br>[-2.368,3.907]	-0.355<br>[-3.511,2.801]	1.704<br>[-2.206,5.614]
T-1 [ref]			
T	-2.157<br>[-5.735,1.422]	-0.503<br>[-4.106,3.100]	-3.058<br>[-7.468,1.351]
T+1	-5.137<br>[-12.797,2.523]	-0.990<br>[-9.141,7.160]	-7.899<br>[-17.561,1.764]
T+2	-7.776<br>[-20.426,4.875]	-2.718<br>[-15.759,10.323]	-13.905<br>[-29.062,1.252]
T+3	-13.629<br>[-30.851,3.593]	-6.692<br>[-23.566,10.182]	-19.937<br>[-40.259,0.386]
T+4	-17.478<br>[-38.647,3.692]	-9.772<br>[-29.953,10.409]	-23.415<br>[-48.735,1.905]
T+5 or after	-35.804*<br>[-64.335,-7.273]	-23.689<br>[-49.626,2.248]	-36.843<br>[-75.237,1.552]

Table 1g. All maternal disorders

	(1)<br>+State & year FE	(2)<br>+Covariates	(3)<br>+Time trends
T-5 or before	-16.728<br>[-142.741,109.285]	-2.452<br>[-67.861,62.957]	23.694<br>[-40.512,87.900]
T-4	-38.931<br>[-131.333,53.471]	-28.777<br>[-68.082,10.528]	-5.466<br>[-34.217,23.286]
T-3	-39.396<br>[-124.233,45.441]	-26.325<br>[-62.762,10.112]	-8.703<br>[-29.793,12.386]
T-2	1.888<br>[-7.546,11.322]	-3.507<br>[-12.823,5.808]	1.926<br>[-11.408,15.260]
T-1 [ref]			
T	-4.133<br>[-15.543,7.276]	-0.709<br>[-11.398,9.981]	-8.276<br>[-23.487,6.935]
T+1	7.405<br>[-32.088,46.898]	10.539<br>[-20.350,41.429]	-23.585<br>[-59.127,11.957]
T+2	9.740<br>[-46.947,66.427]	10.789<br>[-38.210,59.787]	-42.638<br>[-98.671,13.395]
T+3	-5.151<br>[-73.959,63.656]	2.298<br>[-59.036,63.631]	-66.459<br>[-143.595,10.677]
T+4	-24.212<br>[-108.789,60.366]	-9.920<br>[-85.881,66.042]	-86.574<br>[-189.556,16.408]
T+5 or after	-109.870<br>[-290.836,71.095]	-30.798<br>[-158.569,96.974]	-141.074<br>[-336.646,54.498]

Notes for Tables 1a – 1g: Point estimates from linear models are shown. 95% confidence intervals are also shown, derived from standard errors that are clustered by state. A MW event is defined as a change of $1 or more. Outcomes are age-standardized incidence of maternal conditions per 100,000 of the female population. Column 1 is unadjusted, column 2 is adjusted for state and year fixed effects, column 3 additionally adjusts for state covariates (whether the state has expanded Medicaid, the state EITC rate, the TANF benefit level for a 3-person family, the proportion of families eligible for food stamps, the state gross product, and the state population, and the number of births), and column 4 additionally adjust for state-specific time trends. EITC=earned income tax credit. TANF=Temporary Assistance for Needy Families. $^{*}p < 0.10$, $^{**}p < 0.05$, $^{***}p < 0.01$.

Appendix Table 2. Sensitivity analyses

	Maternal hypertensive disorders	Abortion and miscarriage	Ectopic pregnancy	Obstructed labour and uterine rupture	Maternal haemorrhag e	Maternal sepsis and other maternal infections	All maternal disorders
Panel A. Prevalence measures							
Point estimate	-15.8***	-0.9	-0.0	0.6	-3.9*	-5.5	-25.4***
95% CI	[-26.3,-5.2]	[-2.3,0.5]	[-0.1,0.1]	[-0.4,1.7]	[-7.4,-0.4]	[-11.4,0.4]	[-42.0,-8.8]
N	1428	1428	1428	1428	1428	1428	1428
Panel B. Impacts of $0.75 or more							
Point estimate	-42.7*	-46.3	2.2	38.5	-17.8	-56.6	-122.8
95% CI	[-81.1,-4.4]	[-189.2,96.6]	[-10.9,15.3]	[-43.0,119.9]	[-44.1,8.5]	[-121.8,8.6]	[-371.8,126.2]
N	1428	1428	1428	1428	1428	1428	1428
Panel C. Year range from 2000-2019							
Point estimate	-68.7***	61.7	7.2	7.5	-1.7	-11.9	-5.9
95% CI	[-111.4,-26.0]	[-23.0,146.3]	[-0.9,15.3]	[-50.5,65.6]	[-17.7,14.3]	[-67.7,43.8]	[-157.3,145.5]
N	1020	1020	1020	1020	1020	1020	1020
Panel C. Year range from 2010-2019							
Point estimate	35.0	152.9	3.8	-21.9	20.5	-17.8	172.5
95% CI	[-22.8,92.8]	[-46.9,352.8]	[-8.1,15.7]	[-100.0,56.1]	[-17.5,58.4]	[-96.7,61.1]	[-165.0,510.0]
N	510	510	510	510	510	510	510

Notes: Models are fully adjusted for state and year fixed effect, state covariates, and state-specific time trends. 95% confidence intervals in brackets. * $p < 0.05$, ** $p < 0.01$, *** $p < 0.005$

Appendix Table 3. Point estimates and 95% Confidence intervals for alternative heterogeneity-robust estimators, as compared to two-way fixed effects model

Time since MW event	de Chaisemartin-D'Haultfoeuille		Sun-Abraham		Callaway-Sant'Anna		TWFE OLS	
	Est	95% CI	Est	95% CI	Est	95% CI	Est	95% CI
-4	1.17	(-0.91 - 3.25)	3	(-5.15 - 11.15)	18.62	(-52.22 - 89.47)	4.22	(-3.4 - 11.84)
-3	2.73	(-1.56 - 7.01)	2.11	(-3.24 - 7.46)	5.85	(-58.93 - 70.63)	2.93	(-2.48 - 8.34)
-2	3.56	(-3.38 - 10.51)	1.44	(-1.22 - 4.1)	5.84	(-62.93 - 74.6)	2.05	(-0.67 - 4.77)
-1	4.92	(-5.22 - 15.06)						
0	0.00	(0 - 0)	-2.86	(-5.48 - -0.24)	1.3	(-66.04 - 68.64)	-3.59	(-6.29 - -0.89)
1	-1.83	(-4.42 - 0.76)	-6.28	(-11.92 - -0.63)	-6.91	(-56.72 - 42.9)	-8.05	(-14.46 - -1.63)
2	-4.45	(-10.28 - 1.38)	-10.19	(-18.9 - -1.47)	-3.77	(-62.09 - 54.55)	-13.19	(-22.87 - -3.51)
3	-7.06	(-15.88 - 1.76)	-13.21	(-24.71 - -1.71)	-24.7	(-87.56 - 38.16)	-17.91	(-30.55 - -5.28)
4	-10.28	(-21.51 - 0.96)	-15.68	(-29.76 - -1.6)	-27.38	(-127.06 - 72.29)	-22.04	(-36.99 - -7.1)
